# A Review of Biomaterials and Scaffold Fabrication for Organ-on-a-Chip (OOAC) Systems

**DOI:** 10.3390/bioengineering8080113

**Published:** 2021-08-06

**Authors:** Luana A. Osório, Elisabete Silva, Ruth E. Mackay

**Affiliations:** 1 Department of Mechanical, Aerospace and Civil Engineering, Brunel University London, Uxbridge UB8 3PH, UK; ruth.mackay@brunel.ac.uk; 2 Department of Life Science, Brunel University London, Uxbridge UB8 3PH, UK; Elisabete.Silva@brunel.ac.uk

**Keywords:** organ-on-a-chip, scaffold, tissue engineering, biomaterials, additive manufacturing

## Abstract

Drug and chemical development along with safety tests rely on the use of numerous clinical models. This is a lengthy process where animal testing is used as a standard for pre-clinical trials. However, these models often fail to represent human physiopathology. This may lead to poor correlation with results from later human clinical trials. Organ-on-a-Chip (OOAC) systems are engineered microfluidic systems, which recapitulate the physiochemical environment of a specific organ by emulating the perfusion and shear stress cellular tissue undergoes in vivo and could replace current animal models. The success of culturing cells and cell-derived tissues within these systems is dependent on the scaffold chosen; hence, scaffolds are critical for the success of OOACs in research. A literature review was conducted looking at current OOAC systems to assess the advantages and disadvantages of different materials and manufacturing techniques used for scaffold production; and the alternatives that could be tailored from the macro tissue engineering research field.

## 1. Introduction

Research and development in the pharmaceutical and chemical industries have encountered barriers since their conception with regards to the development and selection of appropriate pre-clinical test models [1]. Pre-clinical testing relies on animals or animal-derived models, which are costly, have significant ethical implications and are not accurate representations of human physiopathology [2]. Thus, it is imperative to create accessible animal-free technology and techniques with enhanced predictive power, which allow the detection of unsuccessful or toxic candidates in the early stages of research [2].

Traditional alternative systems to animal models such as 2D/3D cellular cultures are simple, low cost and allow reproducible set-up; nevertheless, they still possess inherent limitations such as an inability to emulate both organ and system-level functions. Significant progress has been made in developing and validating robust/reliable alternative test systems. This has been driven by an increasing acceptance of regulators and industry to commit to the three Rs of animal usage in research (replacement, reduction and refinement) [3].

**Figure 1:**
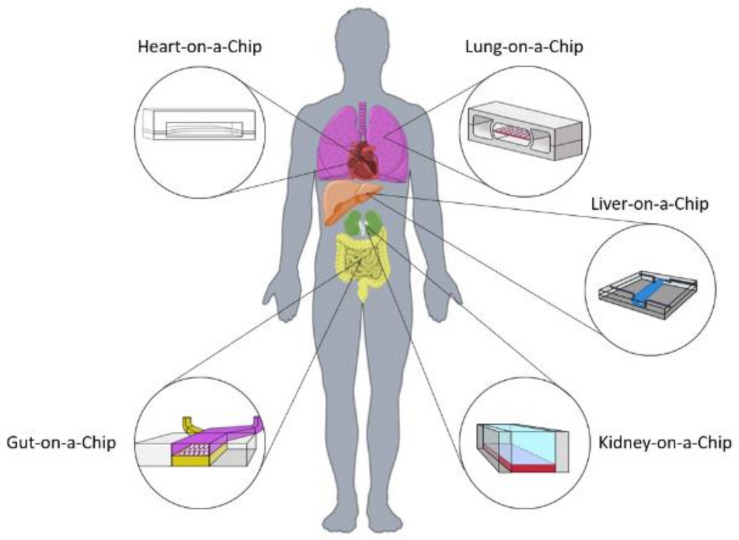
Human-on-a-Chip Scheme [9,10,11,12,13].

For the past 25 years, new concepts of micro-engineering models in the early health technology assessment (HTA) stage [4] have emerged, similar to the microfabrication of versatile microfluidic chips [5]. Organs-on-a-Chip systems (OOAC) are biomimetic systems with µm-scale fluidic channels that aim to recapitulate the complex physiopathology of living human organs by providing a 3D environment susceptible to mechanical, electrical and chemical cues that are key to the architecture and phenotypic changes in tissues [6]. Moreover, OOAC presents a highly competitive advantage with homologous technologies since their size generates small samples with high degrees of functionality and complexity whilst reducing the need for a large number of reagents or significant analysis time [7]. The concept of an integrated Human-on-a-Chip (Figure 1) based on the connection of different validated OOACs devices could be used as a potential future alternative to animal models in pre-clinical trials for analysis of drug side-effects and patient-specific response. This model not only would cut cost and time in pre-clinical testing but would also lead to the replacement of animal subjects as a whole in the pharma and cosmetic industry [8].

The OOAC system consists of a microfluidic chip with chambers and channels where cells are seeded into an appropriate matrix or scaffold. The heterotypic culture of cells (i.e., the culture of different cell types or different cell subpopulations) in a microfluidic chip should, at a simplistic level, be able to emulate the different layers of tissue to replicate the organ’s functionality, vascular system and to provide a structural extracellular matrix (ECM). Co-culture systems aim to explore how cells react to different stimuli such as chemotaxis and mechanical cues that affect tissue function and molecular pathways [14].

To allow a successful culture of cells in 3D and, consequently, replicate physiological tissue architecture, naturally derived and synthetic polymeric scaffolds are included in OOAC systems. These scaffolds should be chosen based on the demands of the specific cellular tissue since a variety of materials and techniques are used to alter the scaffold characteristics [15].

State-of-art commonly used OOAC scaffolds include poly(dimethylsiloxane)—PDMS—and Matrigel^TM^, but both materials pose some challenges in organotypic cultures of human cells. The Lung-on-a-Chip system, for example, uses microporous elastomeric membranes of PDMS as its flexible properties allow the emulation of the mechanical stretch these cells undergo in the lung environment [9]. Nevertheless, this is a non-degradable material that does not contribute to the formation of natural ECM. As an alternative, Matrigel^TM^ is an animal-derived gel-like scaffold that contains growth factors and hormones, promotes cellular attachment and provides a 3D architecture for tissue formation [15]. However, due to its origin, there is significant batch-to-batch composition variability, which affects experimental results reproducibility [16]. These examples show how scaffolds currently used within the OOAC research field are still flawed and can lead to the failure of these systems as reliable pre-clinical models.

This paper aims to provide a summary of scaffold requirements, review different materials and manufacturing techniques that can be used to produce the optimal scaffold according to the OOAC specifications. The paper will analyse the advantages and disadvantages of currently used methods and how macro-tissue engineering techniques could transcend into the OOAC research field.

## 2. Scaffold Requirements

The extracellular matrix is a 3D agglomeration of extracellular molecules such as collagen, enzymes and glycoproteins produced by cells, which are responsible for the structural architecture of in vivo connective tissues. Cell behaviour is modulated by the dynamic role of mechanical and spatial signals from the ECM [17].

The ECM is a molecular network that has proteins, glycosaminoglycan and glycoconjugates as its major components [18]. It is responsible for the regulation of cellular migration, wound healing, cellular anchoring, and the differentiation process. The organisation of the ECM consists of the interstitial matrix—an amorphous gel made of collagen, elastin and fibronectin—and the basement membrane—denser and less porous than the interstitial matrix, highly organised tissue found in epithelial and endothelial tissues. In tissue engineering, the ECM is used as a scaffold and to induce differentiation of MSCs [19].

When creating an OOAC system, one must consider the type of tissue they aim to recapitulate since the scaffolding characteristics will be different. For example, many connective tissues such as articular cartilage and the intervertebral disk have large amounts of water and proteoglycans [20]. While, for instance, mammary epithelial tissue ECM presents large quantities of fibronectin, tenascin, laminin, among other molecules, that largely influence cell behaviour [21]. Effective 3D scaffolds need to be porous to allow media perfusion and cell proliferation, biocompatible with the specific cell type used and made of biodegradable materials thus that cells may replace them with their natural ECM [22,23].

Creating organ emulating scaffolds is a challenge since many variables must be taken into consideration, including biocompatibility, biodegradability, pore size and suitable mechanical properties [24].

A plethora of biomaterials and manufacturing techniques have been rapidly developing. However, each biomaterial still presents limitations to achieve optimal scaffolds for OOAC systems [25].

### 2.1. Biocompatibility

When manufacturing a scaffold, biocompatibility must be ensured since cellular tissue has a natural tendency to reject external materials if they fail to provide the specific nutritional and biological conditions for cell maintenance [26]. Cell culture requirements include temperature, pH, oxygen and carbon dioxide levels, and the use of specific nutritional mediums for the specific cell line. Temperature affects growth and production processes, and it ranges between 36–37 °C for most human and mammalian cell lines. The cell culture medium must provide a continuous supply of oxygen to the cell through diffusion on a liquid surface. A total of 4–10% carbon dioxide is also used on most cell culture experiences since it assists in balancing pH levels as a buffer in the growth medium. The majority of mammal cell lines grow at a pH of 7.4, with some fibroblast lines being reported to grow in slightly more basic pH (7.4–7.7) [27,28]. The cell culture medium can be naturally derived or artificially manufactured. Natural medium includes biological fluids (plasma, serum, etc.), tissue extracts or clots (coagulants or plasma clots) [29]. Artificial medium is prepared by adding nutrients such as vitamins, salts, O_2_ and CO_2_ gas phases, cofactors, among others [30]. The composition of the medium varies according to its purpose, for example, indefinite growth or specialised functions [29]. For the OOAC system to be successful, these requirements must still be met thus that the tissue may grow healthy and emulate physiological conditions [31].

Scaffolds must allow cell adhesion whilst providing a biologically functional environment that allows cell migration [24]. The biomaterial utilised must be safe and not induce cytotoxicity. Thus, OOAC systems that house immune system cells must not provoke an inflammatory response or exacerbate immunogenicity [32].

Moreover, robust cell adhesion is crucial for the successful cell proliferation process, which is divided into three steps—cell attachment, cell spreading and focal adhesion between cells and the scaffold surface [33]. Cellular differentiation is affected by the microstructural and physicochemical properties of the scaffold since they impact protein adsorption. Thus, altering the surface and topography of the scaffold makes it tunable to protein adsorption and cell adhesion according to its function [34].

With the growing interest in technologies such as microfluidic chips for research, the development of biocompatible materials has exponentially increased [35].

### 2.2. Biodegradability

The main goal of tissue engineering is to provide a structure where cells can grow and ultimately replace the scaffold with their own natural ECM. Hence, manufactured scaffolds must be biodegradable [24]. The use of synthetic biodegradable polymers has been significant in tissue engineering applications since it can improve mechanical properties for tissue growth. A suitable biodegradability rate must be inversely proportional to the production of natural tissue by the cells [36]. Biodegradation kinetics also play a role in the release of drugs for tissue manipulation. An example of this can be seen in long-term therapies where slow degradation kinetics scaffolds such as PLA, PGA and PLGA to name a few, are used to perform a controlled drug release [37]. The degradation of the manufactured scaffolds results in by-products over time, which cannot be toxic to the cells and should be easily flushed out of the scaffold [32]. For example, it has been observed that PLA, PGA and their co-polymers to be related to acidic by-products, which affect the pH of the environment due to the production of lactic and glycolic acid resultant from the hydrolysis of these scaffolds [38,39]. The biodegradation rate of the scaffold is dependent on the scaffold’s specifications (composition, microarchitecture and mechanical properties), abiotic environmental factors (temperature, pH, salinity) and biotic factors (cell type cultured, microbiome) [40]. Furthermore, studies have proven that in vivo has an accelerated biodegradation rate when compared with to vitro models due to factors such as lack of immune system and hydrolytic/enzymatic properties [41]. Moreover, when comparing the degradation rate in static versus microfluidic models, it has been observed that the insertion of flow significantly changes the degradation conditions. The flow also produces a mechanical load onto the scaffolds and consequent shear stress, which leads to weight loss by degradation of the scaffold, further the flow perfusion increases the surface area to degradation. For example, Ma et al. reported the highest porosity in PLGA scaffolds in a micro-channel condition when compared with incubator static and incubator shaking conditions, with the latest recording 0% porosity between day 21 and day 28, while the micro-channel conditioned scaffold maintained porosity at 8%. [42]. There is a gap in research concerning degradation rates in microfluidic devices since many factors can change the time a scaffold takes to degrade, such as the type of OOAC model that is being used, the physiological function of each cell type [43], the flow rate, different types of reagents used in the experiment, porosity and pore size and material chosen. Furthermore, the timeline of degradation of a certain scaffold is not comparable to in vivo and static in vitro because of the reasons explained beforehand.

OOAC systems that aim to reproduce the symbiosis between cellular tissue and the microbiome, such as the Gut-on-a-Chip must also consider the degradation rate of the seeding scaffold when adding different strains of bacteria to emulate the in vivo environment [44]. Enzymes produced by bacteria have been shown to significantly accelerate the catalytic chemical degradation of scaffolds when compared to systems without a microbiome present [40].

### 2.3. Mechanical Properties

Mechanical forces are transduced into biochemical cues that regulate cell communication, active phenotypical changes, and cellular function accordingly. In vivo tissue barriers are continuously subjected to cyclic mechanical strain [45].

Scaffolds used in OOAC systems must mimic the conditions of the cellular tissue or organ they aim to replicate by maintaining mechanical integrity, thus allowing the proper physiological function of the tissue [46,47]. Moreover, the chemical composition, construct and function of the natural in vivo ECM must be replicated [48]. Natural tissues contain structural proteins like collagen and fibronectin. These proteins are commonly used to functionalise different scaffolds and improve their characteristics [49].

Mechanical strength is a property critical for a scaffold to be used successfully in cell culture [50]. Tests, such as tensile strength, compressive stress and wettability, are commonly performed to assess if these properties are within the suitable range. Wettability assays give an insight into the scaffold surface roughness and porosity as well as biocompatibility [51]. Tensile strength tests allow the study of the scaffold viscoelastic properties [25]. As tissues are viscoelastic, they show signs of creep as mechanical forces cause molecules within the ECM to rearrange; when the force is removed the tissue returns to its original shape. Young’s modulus (E) gives an insight into the elasticity of the material; it is the ability of the material to undergo a change in length when under either a tensile or compressive stress.

Biomaterial properties change according to manufacturing techniques and conditions of the surrounding environment, thus it is critical to evaluate each scaffold individually [25].

The mechanical properties of the scaffold should be chosen to match those of the tissue that is being formed. The arterial wall has a Young’s modulus of 1 MPa. Researchers trying to mimic this in an OOAC use a PDMS scaffold with E = 705 kPa [52]. Lung tissue is less stiff and shows E = 3.4 kPa in uniaxial tension [53], therefore, a scaffold with similar properties would give optimal emulation of the organ. Previously groups tended to PDMS as the scaffold of choice when developing Lung-on-a-Chip models [9]; however, due to its high Young’s modulus, hydrogels are becoming more common as they can mimic the stiffness of the lung ECM. Huang et al. recently showed a GelMA based scaffold with a modulus of 6.23 ± 0.64 kPa [54].

Cells and tissues within OOAC devices undergo a range of mechanical stimuli, including laminar, pulsatile, and interstitial flow along with tensile and compressive forces, a good review of methods is written by Kaarj and Yoon [55]. Forces can be applied to scaffolds in OOAC devices to actuate tissue structures. These are in the order of µN to mN depending on the dimensions of the scaffold. These forces have a significant role in tissue development. It has been found that in the basal membrane of the lung, is thinner and, therefore, less stiff than the surrounding ECM. These lower stiffness sections are where epithelial buds form [56]. Shear stress has been shown to affect the differentiation of stem cells [57]. Mechanical stimulation can alter the expression of proteins from cells whilst the ECM stiffness regulates cell behaviour [56].

### 2.4. Scaffold Architecture

The tissue framework demands an interconnected pore network and high porosity to guarantee cell proliferation and continuous flow of the medium. The network provides nutrients, gas exchange and an elimination route of cellular waste and scaffold degradation by-products [24]. As such, the architecture and porosity of the matrix must be finely balanced to ensure the facilitation of protein exchange between cells through the interconnected pores, without, however, compromising the mechanical integrity and stability [58].

The mean pore size and surface area of the scaffold are intrinsically connected to the ligand density. Therefore, an optimal scaffold architecture not only entails large pore sizes for cell migration but, at the same time, small enough pores that an adequate high specific area surface is available with a low ligand density [59,60].

For example, in 2016, the effect of different pore sizes and porosity on a chondrocyte seeded scaffold was studied by analysing cell proliferation, viability and cell-specific creation of cartilaginous ECM. The results demonstrate that cell density increased up to 50% with an increase of pore size and porosity associated with bigger surface area for migration and mass gas/nutrients exchange within the scaffold. Moreover, the production of GAG (glycosaminoglycan) and collagen-specific constituents also increases proportionally with pore size. Nevertheless, it was reported a decrease by 40% of metabolic activity and ECM synthesises with pore size increase. These results imply that chondrocytes synthesise less matrix when the pore size is bigger. This study shows how important the scaffold architecture is to the behaviour of cell cultures and that different considerations must be undertaken to understand what the needs for a specific cell line are when choosing the material/manufacturing technique to produce a scaffold [61].

Furthermore, surface topography is incredibly important to regulate cell fate. In vivo actin cytoskeleton remodelling controls the mechanical homeostasis of cells, thus it is imperative that this function is recapitulated in synthetic scaffolds for OOAC devices through the design of topographic and mechanical indications on the surface of the scaffold [62]. For instance, cell alignment can be affected by altering protein adsorption. Moreover, on PLGAA scaffolds, the use of type-I collagen meditates attachment [63].

### 2.5. Manufacturing Technology

The manufacturing technique used to create the scaffold will influence both the mechanical properties and architecture. It must be cost-effective and permit scale up to batch fabrication for the research industry [24]. Scientific advances have allowed the creation of manufacturing technologies such as electrospinning [26], 3D printing [64] and injection moulding [65], among others, which enable the fabrication of complex constructs. Fabrication standards must be established to guarantee the different scaffolds can be used for pre-clinical OOAC system testing [35].

### 2.6. Scaffold Integration in OOAC System

OOAC systems have a chamber that is fed by an inlet for media and an outlet for media waste. The chamber not only plays the role of housing the scaffold and tissue but is also engineered to mimic mechanical cues if necessary. For example, the chamber of the Lung-on-a-Chip undergoes cyclic mechanical stretching through vacuum manipulation to recapitulate breathing movements [66].

The integration of the scaffold within the chamber is crucial for the functioning of the chip as a pre-clinical model. The type of scaffold being used—hydrogels or stiffer materials for scaffolding—must be taken into consideration when designing the chip system and will dictate how it can be applied to the system [67].

#### 2.6.1. Hydrogels

Hydrogels are 3D polymer frameworks that contain high volumes of water [68]. These materials are permeable due to the affinity of the polymer to a solvent’s molecule [69]. OOAC devices are micro-scaled, which means methodologies must be adapted to accommodate the filling of the device with this type of scaffold. Approaches vary between fabricating a scaffold with hollow channels or creating channels from the material itself [70,71].

Virumbrales-Muñoz et al. used a microfluidic device to study the capability of TNF-related apoptosis-inducing ligand (Trail) to kill tumour cells by using Collagen scaffolding. The hydrogel was placed on top of the inlet and polymerised at 37 °C, followed by culturing of endothelial cells [72]. This is one of the many examples where a hydrogel is delivered to fill a channel or chamber through an inlet and held in place by the surface tension, which undergoes subsequent curing. Nevertheless, this simple method comes with limitations such as the lack of cell alignment [73] and the lateral flow, which feeds the cells might be insufficient depending on the thickness of the scaffold since hydrogels thicker than 200 µm can lead to cell necrosis from the lack of oxygen delivery [74].

Another method is the fabrication of microchannels in hydrogel parts, as referred to above. A master mould created using photolithography, sacrificial or physically removable moulds are frequently used to create microfluidics within the chosen hydrogel [75]. This is a straightforward technique referred to as replica moulding. Nevertheless, it still presents challenges such as the use of stiffer materials that, to structural integrity, affect the viability of cellular encapsulation since they have high material density. Moreover, deformation of the created microstructures in soft gels has been reported [67]. Bioplotting has been vastly used due to its ability to create 3D structures with a specific design [76]. Both bioplotting and replica moulding techniques can be used to create microfluidic devices from hydrogels [67].

#### 2.6.2. Additive Manufacturing Derived Scaffolding

Additive manufacturing derived scaffolds such as electrospun and 3D printed scaffolds require different approaches concerning their integration within an OOAC system. Static systems protocols include the peeling of the membrane from the collector, cutting it to the desired shape, followed by the placement of this membrane in the culturing chamber after proper sterilisation. Nonetheless, the simplicity of this procedure derives from the lack of shear stress, waste removal and gradient control characteristic of OOAC systems [67].

Different methods have been developed to introduce scaffolds into microfluidic chips. One of these methods is the lateral flow model used by Pimentel et al. where certain areas of the membrane are occluded to create a hydrophobic zone around the channels [77]. The disadvantage of this method is that it is not appropriate for cell lines that need shear stress [67].

Another method of integration of the scaffolds into the OOAC was developed in 2016 by Chen et al., which was denominated by dynamic focusing electrospinning technology, which enables the spinning of a scaffold directly onto the fluidic chamber. This method enables the introduction of flow through the tissue but requires a channel wider than 1 mm [78].

Modular integration of the manufactured scaffold in the microfluidic system was reported by Chen et al. in 2018, where the membranes are cut into the dimensions of a specific chip. Cells are then seeded onto the scaffold and then inserted onto the microfluidic chamber, followed by the introduction of flow to the system [79]. This method presents advantages such as the ability to remove the cellular cultured inserts, for example, in case of contamination or for imaging, from the system itself [67].

## 3. Biomaterials

The chemical composition of different materials—both naturally derived and synthetic polymers—plays a vital role in the enhancement of the scaffold’s properties mentioned in the previous section, which are critical for the growth of cells in the manufactured scaffolds [35].

### 3.1. Naturally-Derived Materials

Natural polymers are obtained from nature—plants, animals, insects and even humans. These materials provide reliable scaffolds since their mechanical and chemical properties are identical to those in the natural tissues that we aim to replicate, allowing cells to easily proliferate and differentiate in the OOAC system [35].

#### 3.1.1. Matrigel

Matrigel^TM^ has been widely used in the Tissue Engineering field for the past 40 years. This basement-membrane matrix is extracted from the Engekbreth-Holm-Swarm mouse’s sarcoma, which produces large amounts of ECM [80]. It is one of the most sought-after animal-derived materials for the development of three-dimensional cell cultures since it provides a gel-like scaffold that enables cells to form more complex and accurate structures than two-dimension planar cell cultures [15]. It promotes cell adherence due to its primary composition of over 1000 ECM proteins [81], including—laminin (~60%), type IV collagen (~30%), entactin (~8%) and the heparin sulphate proteoglycan perlecan (~2–3%) [80]. These proteins allow for the existence of microenvironments of soluble growth factors—transforming growth factor (TGF) family peptides (for example, TGFβ) and fibroblast growth factors (FGFs) [81]—along with hormones among other major molecules [16].

One successful example of the use of Matrigel^TM^ in OOAC platforms is the Liver-on-a-Chip system. The Liver-on-a-Chip system was developed as a method to increase the prediction rate of Drug-Induced Liver Injury. The membrane is deposited in between two cell layers [10]. Three-dimensional cultures within Matrigel^TM^ have been proven to be significantly more sensitive to drugs rather than two-dimensional cultures and present cellular structures that closely resemble the in vivo tissue [82]. Moreover, Matrigel^TM^ has been embedded with hepatocytes on a microfluidic chip by Jang et al. since it maintains cellular morphology by preventing cellular differentiation and polarization [83].

Nonetheless, the use of Matrigel^TM^ has been shown to be restrictive by several proteomic analysis studies due to the batch-to-batch composition variability. Some factors responsible for this variability include protein and endotoxin concentrations [84] that can impact the formation of 3D structures, as well as the presence of numerous growth factors that can affect immunoassays [81] Further, the existence of xenobiotic contaminants, which act as confounding factors, lead to confounding results and an inconclusive understanding of which signals promote cells functioning [85].

Consequentially, the composition inconsistency alters the mechanical properties between batches. For example, Young’s modulus, has been shown to be low at <0.2 kPa [86,87,88]. Furthermore, the animal-derived nature of Matrigel^TM^ can influence cellular behaviour [89].

Therefore, there is a need to replace Matrigel^TM^ with a material that can recreate a three-dimensional environment whilst still allowing cell adherence, cell signalling and mechanical cues, that do not have a variable batch to batch protein composition [16].

#### 3.1.2. Collagen

One of the most predominant proteins in the ECM is collagen since it accounts for approximately 30% of the proteins within a mammal [90]. Its fibrillar structure contributes to the extracellular scaffolding of natural tissue, maintaining the structural integrity of the tissue [91]. Not only does collagen control morphology, adhesion and differentiation of the cells, but it has also been found to have low immunogenicity, good biocompatibility, porous structure leading to good permeability [92], and it is more biodegradable when compared to other scaffolds [93].

Collagen can be extracted from different species, including rat, bovine, porcine and fish, among others, and its origin influences the solubility of the scaffold structure [94,95]. This scaffold has been used for decades to regenerate damaged tissue; however, its use in 3D culture has increased in recent years due to its tunable properties [96]. The human colon has a Young’s modulus of E = 0.63 ± 1.25 MPa [97].

The Gut-on-a-Chip incorporates a collagen scaffold to emulate the shape and density of human intestinal villi. This hydrogel scaffold was chosen because it allows to closely emulate the native ECM microenvironment of the gut, inducing cell differentiation while maintaining integrity and barrier function. This scaffold is seeded with Caco-2 cells and results in a uniform monolayer across the surface of the villi [98]. The system was developed as a solution to the limitations of 2D in vitro models, with the goal of understanding the complex process of absorption and metabolism of drugs in vivo [99]. It has proven difficult for scientists to acquire accurate results for Young’s modulus of collagen with a range from 0.13–9.1 kPa [88,100].

Nonetheless, collagen manufactured scaffolds lack mechanical strength and structural stability when hydrated, which limits their application. To overcome this limitation, collagen scaffolds are often mixed with other materials, or intermolecular cross-linking can be used to improve the mechanical stability of the structure. Moreover, further biochemical factors can be included in the scaffold to enhance cellular response [101]. The polymerization reaction is dependent on temperature, pH, ion chemistry and monomer concentration, which affects its inherent properties. For example, increasing the pH value results in decreased fibre diameter and pore size and stiffer gel matrix [102].

#### 3.1.3. Chitosan

Chitosan is the second most common amino polysaccharide that is obtained as a deacetylated derivate of chitin. Chitin is found abundantly in the exoskeleton of crustaceans and insects [103]. Chitosan has a similar structure to glycosaminoglycans and has been used in the pharmaceutical industry since the early 1990s owing to its immense beneficial properties such as biocompatibility, biodegradability, antibacterial and antifungal activity; it is a polycationic polymer and shows permeation enhancement [104]. Chitosan hydrogels have a low interfacial tension with water and biological fluids [105] and the capability to absorb water without compromising structural stability. The mechanical properties of this biomaterial are dependent on the charge and degree of crosslinking. For example, enhancing the crosslinking leads to increased stiffness and Young’s modulus [106]. Its crystalline nature allows it to be processed using different methods to create different scaffolds types, including gels, nanofibres and sponges [107]. Moreover, chitosan membranes can be responsive to light, pH, temperature and ionic concentration [108].

For example, collagen-chitosan hydrogels were used by Chiu. et al. as scaffolds for the vascular OOAC system to achieve angiogenesis with endothelial cell proliferation. The positively charged nature of the chitosan was used to induce the sustained release of negatively charged encapsulated Tβ4, which is a protein that is used to increase cell density in scaffolds in this specific system. [109].

Nevertheless, chitosan and chitin scaffolds have limitations as mechanical tests have shown them to be mechanically weak and unstable. These vary according to chitosan weight percentage, gelation method and degree of acetylation; one study showed results from 5–2500 kPa [110] by altering these factors. Hence, polymeric blends are used to enhance these properties [107].

#### 3.1.4. Alginate

Alginate or alginic acid is an anionic polysaccharide found in brown algae. The production cost of this polymer is reduced by marine extraction from algae, and it can also be synthesised through microbial fermentation [111]. Mechanical and chemical properties of alginate may vary according to the seasonal growth conditions of the source [112]. Algae derivates have abundant G blocks content, while bacterial derivates possess high concentrations of M blocks content. Currently, there are more than 200 alginates being produced [113]. It has been considered biocompatible with human tissues both in vitro and in vivo. However, the purity of alginate can impact this factor [114].

Alginate nanofibres have shown good potential as a tissue engineering scaffold for skin, bone, cartilage, and liver cell types. Nevertheless, the manufacturing of these scaffolds is still associated with many challenges, such as successful electrospinning of pure alginate nanofibres due to the strong inter and intramolecular hydrogen bonding between the stiff molecular chains [115]. Hence, composites with other polymers or cross-linking methods are created to improve the ability to be electrospun [116].

The viscosity of alginate solutions increases with the decrease of the pH, reaching its maximum viscosity at a pH ranging between 3–3.5. The molecular weight of commercially sold alginate is between 32,000–40,000 g/mol [117]. Altering the molecular weight of the solution can improve the mechanical properties of the hydrogel [118]. Without additives, the hydrogel has been shown to have a Young’s modulus of 0.2–1.3 kPa whilst later studies have shown higher values up to 6 kPa as the weight percentage of alginate is increased [88,119]. Alginate is not naturally degradable in mammals as they lack the enzymes to cleave the polymer. Hence, the polymer can be cross-linked with divalent ions to increase its solubility [117]. Moreover, alginate lacks the ability to bind to mammal cells, which is vital for the regulation of cellular interactions between scaffold and tissue. Consequently, peptides such as RGD are used as adhesion ligands to increase cell viability [120].

Alginate membranes have been used in microfluidic devices with different cell types (cardiac tissue, liver, and hepatocyte spheroids) for cell encapsulation [121] as sacrificial material for vascular network patterning [122] and for drug testing models [123]. This scaffold has been reported to emulate the 3D ECM of many soft tissues both chemically and physically. However, this material still presents its challenges when it comes to using it on microfluidic devices, such as its flexibility and controllable formation [121]. Besides, studies have reported poor cell attachment to alginate scaffolds because of the inability of human cells to attach to negatively charged surfaces [123].

#### 3.1.5. Cellulose

Cellulose is the β-(1→4)-linked polymer of D-glucose that has a structural role as a load-bearing element in the cellular wall of plants. It is the most common naturally derived molecule in the environment [124]. Cellulose type I is the crystalline form of natural cellulose and has two existing polymorph forms with different origins—cellulose I_α_ and cellulose I_β_. Cellulose I_α_ is a single chain triclinic unit mostly present in bacteria-derived cellulose. On the other hand, cellulose I_β_ has a double chain monoclinic structure, which can be retrieved for instance, from cotton and wood [125]. The microfibrils range from 10–30 µm depending on the cellulose sources [126].

Bacterial cellulose (BC) derived from *Acetobacter xylinum* is abundantly used in the pharmaceutical industry as a scaffold. It is insoluble in water and is degraded by microbial and fungal enzymatic activity [127]. Cellulose nanofibrils create a network with high mechanical strength, a high level of water retention [128] and show high hydrophilicity, which is optimal for cellular growth and proliferation in three-dimensional scaffolds [127]. BC network densities are tunable biomaterials that can be modified by varying the cell culture conditions [129]. The Young’s modulus in air-dried BC sheets has been shown to be as high as 16.9 GPa, with an elongation of 1.7% and tensile strength of 256 MPa [130].

Cellulose nanofibres are transparent, which is beneficial for microscopy analysis of cell morphology throughout the experiment [131].

Shin et al. developed a microfluidic platform that used a cellulose nanofibre to understand the role of cisplatin in the death of lung cancer cells and test the lethal dose of anticancer drugs; this in vitro model was proven to be effective in the study of cell behaviour, cell–cell interactions and chemical toxicity [132].

#### 3.1.6. Gelatin

Gelatin is obtained by hydrolyzing and denaturing animal skin [133]. Its origin makes it extremely biocompatible, biodegradable and non-immunogenic. It has good water-solubility and is readily available from commercial sources for the emulation of human tissue [134]. Whilst gelatin composition is similar to collagen, it is a lower cost polymer and less antigenic [135].

Gelatin has been one of the most promising polymer scaffolds because of its biodegradability [136]. The source of the gelatin and the conditions in which it is extracted dictate the molecular structure and physical properties of the polymer. The Bloom Index is used as a measure of gel strength for gelatin. The Bloom index has higher values when gelatin extraction occurs at lower temperatures making the hydrogel stiffer [137]. The deformation and stress at break increase with the increase of the Bloom Index, consequently, Young’s modulus increases linearly with this parameter [136].

Gelatin hydrogels have been used as a tunable scaffold to emulate the cardiac tissues ECM (Young’s modulus ranging from 10–15 kPa [138]) due to a similar Young’s modulus, that is similar elastic properties when undergoing tension or compression in one direction. These showed higher spare respiratory rates with an improved metabolic function when compared with fibronectin-coated PDMS scaffolds [139]. Moreover, vascular chips were successfully developed with gelatin methacrylate (GelMA) as a vascular model since it is a denatured derivate from the most common protein in the body and contains RGD. It is semi-transparent, allowing UV crosslinking. This study demonstrated that this material lined with HUVECs creates a good barrier function. [140]. GelMA has also been used because of its biocompatibility and low viscosity as a scaffold for a muscle model [141].

### 3.2. Synthetically Derived Materials

To create low-cost enhanced scaffolds, new approaches have been taken by researchers to create new synthetic materials that could replace naturally derived biomaterials without compromising their key requirements.

#### 3.2.1. Poly-(Ɛ-caprolactone)—PCL

Poly-(Ɛ-caprolactone) is a linear synthetic biodegradable polyester, which can be acquired at a low cost, and is extremely versatile for scaffold development [142]. This aliphatic polymer has been approved by the Food and Drug Administration (FDA) for biomedical and tissue engineering applications due to its ideal mechanical and biological properties. Biodegradable polyesters are frequently used in scaffold manufacture: these include PCL and poly-L-lactide [143].

The biodegradability of PCL has been tested and shows large variation dependent on specific parameters, including material properties, scaffold architecture and environmental conditions. Microorganisms have been shown to accelerate the degradation of PCL scaffolds [144]. Nevertheless, hydrolytic cellular mechanisms have since been proven to cause slow degradation of PCL when compared with naturally-derived polymers [145,146].

PCL is a hydrophobic material that is problematic for cellular adherence and interaction [147]. Thus, to functionalise its surface, it is frequently blended with amino acids sequences (RGD, GRGDSP, PSHRN and IKVAV) and peptides such as fibrin [142]. PCL tends to be used as a copolymer as it has stable mechanical properties that can enhance the stability of naturally-derived materials whilst increasing hydrophilicity, cell adherence and viability [148]. The average molecular weight of PCL ranges from 530 to 630,000 Mn/g mol^−1^ [142]. PCL is soluble in most organic solvents such as chloroform, cyclohexanone and 2-nitropropane at room temperature [149]. Scaffolds of bulk PCL have a tensile strength between 25–43 MPa and a Young’s modulus ranging from 330–360 MPa; porous and fibrous scaffolds have lower tensile strength and Young’s modulus dependent upon scaffold architecture [150].

PCL/Polydopamine (PDA) scaffolds have been used to emulate the myocardium in a muscle-inspired microfluidic system aimed at measuring cardiac contractility. The scaffolds resulted in a framework that gave topographical signalling for the formation of anisotropic cardiac tissue [151]. A porous PCL/poly(lactic-co-glycolic acid)—PLGA—microfluidic perfusion membrane was developed to replicate the vasculature network. This scaffold has been used for co-culture of endothelial cells, pericytes, astrocytes and neural stem cells in an ex vivo blood-brain barrier model, with high mechanical strength, biocompatibility and biodegradability [152]. Furthermore, PCL scaffolds coated with collagen gel were used to investigate the cell-cell and cell-ECM interactions in endothelial cells monolayers lining with the purpose of further understanding vascular diseases and cancer metastasis [153].

#### 3.2.2. Poly-(dimethyl-siloxane)—PDMS

Poly-(dimethyl-siloxane) or PDMS is the most widely used silicon-based organic polymer for both the manufacture of microfluidic chips and scaffolds due to its elastomeric properties, cell biocompatibility, gas exchange and optical transparency [7].

PDMS came to the forefront of OOAC systems due to its abundant use in Lab-on-a-Chip systems—an area of analytical science that proceeded OOAC. PDMS was used primarily within academic research due to its low cost and ease of manufacturing using soft lithographic methods [154]. It is optically transparent, which allows real-time analysis of cell cultures without having to destroy the OOAC system; however, it is hydrophobic, which poses a disadvantage for cell adhesion [155]. It is extremely flexible due to its low glass transition temperature (T_g_ −125 °C) [156].

PDMS is known to adsorb small molecules and drugs, which can either be an advantage or disadvantage depending on the chosen application [157]. The Si-O- bond in PDMS is responsible for its thermal and chemical stability [158].

PDMS scaffolds produced by Si, J. et al. have a compressive modulus of 19.69 ± 1.42 kPa and compressive strength of 4.76 ± 0.22 kPa due to its high porosity [159]. The stiffness of the membranes varies according to the type of cell that is cultured and the formation of their focal points [160]. Even though PDMS is largely used as a scaffold for the OOAC systems, it possesses a vital disadvantage, it is not biodegradable, which means it cannot be replaced by natural cell-produced ECM [161]. Moreover, lasting submersion in organic solvents leads to swelling and detachment of the produced layers affecting the surface of the membrane [162]. PDMS is often coated with ECM proteins to increase cell attachment. However, the bond between both can be degraded when exposed to sufficient shear stress from the flow in OOAC systems [163]. The Lung-on-a-Chip takes advantage of the PDMS flexibility to emulate the cyclic loading lung tissue is subjected to. A thin (10 µm) porous PDMS membrane was coated with fibronectin or collagen, human alveolar epithelial cells, and human pulmonary microvascular endothelial cells [164].

In 2018, Quirós-Solano et al. reported cell migration and healthy growth of cells in the porous membranes with HUVEC and MDA cells while using this polymer as a spin-coated scaffold [162].

A Kidney-on-a-Chip device has also been created using PDMS membranes to test the drug metabolism process that influences renal adverse effects. Liver slices were cultured between a polycarbonate and PDMS membrane [165]. Moreover, PDMS membranes coated with ECM were seeded with Caco-2 intestinal epithelial cells to emulate a human Gut-on-a-Chip model [13].

#### 3.2.3. Polylactic Acid—PLA

Polylactic acid is a biodegradable polymer firstly synthesised in 1780 and is acquired from the chemical synthesis of acetaldehyde and carbohydrate fermentation process [166]. PLA’s molecular weight distribution impacts the mechanical, biological and degradation rate [167]. This parameter can be modified by azeotropic distillation and a longer polymerization time [168]. PLA can be dissolved in various solvents, including dioxane, tetrahydrofuran and hot benzene [169]. It is a thermoplastic material that can be manufactured into fibres and films. The mechanical properties vary according to the scaffold manufacturing technique used [170].

PLA can be degraded when its ester bond is exposed to hydrolysis and the degradation rate is dependent on the isomer ratio, temperature of the reaction and structure and size of the scaffold [171].

PLA has numerous distinctive forms, such as poly-L-lactide (PLLA) and poly-D-lactide (PDLA) since lactic acid has chiral nature. This allows the stereochemical structure to be modified in order to change its mechanical and chemical properties [169].

Cell culture chips have been prepared with PLA exhibiting different surface topologies with the aim of housing human mesenchymal stem cells (MSCs) and studying the phenotypic differentiation they undergo. It was shown that smaller and higher density pores on the surface of PLA induced pluripotency of human MSC. PLA membranes that are exposed to mechanical strain are not recommended due to their high Young’s modulus, membranes that incorporate PEG to act as a plasticiser, and lower Young’s modulus still show high values with E = 5–7 MPa [49,172].

#### 3.2.4. Polyethhene Glycol—PEG

Polyethene Glycol or PEG hydrogels are highly biocompatible with natural tissue, and their mechanical properties can easily be manipulated by adjusting weight percentage, molecular chain length and cross-linking density. Cross-linked PEG forms a porous hydrogel that closely emulates the ECM of human tissues [173]. The liquid-to-solid transition for the gelation process can be easily controlled in the presence of cell suspensions [174]. It is an inexpensive scaffold derived from the living anionic ring-opening polymerization of ethylene oxide, and its molecular weight ranges from 0.4–100 kDa, and it is soluble in water [175]. Circulation of small molecules has been reported to be extended without compromising the bioactivity of PEG attached through covalent or noncovalent interaction [176]. Furthermore, PEG is hydrophilic and inert, presents low protein absorption, which is important for the cross-linking and ligands presentation to cells [177]. The polymer concentration, chain length and configuration have been discovered to play a role in the mechanical and chemical properties of PEG, which can easily be engineered in accordance with the tissue to be replicated [178]. Young’s modulus for PEGDA was found to vary from 0.5–1.9 kPa for concentrations of 5–20% (weight:volume), respectively, whilst values up to 700 kPa have been seen by using low molecular weight PEG [88].

PEG hydrogels are FDA-approved and have been vastly used in regenerative medicine as a delivery strategy and as a scaffold. PEG flexibility can be manipulated when using different functional groups and mixed polymerization techniques [177]. However, it is commonly used in combination with other biopolymers, such as hyaluronic acid, fibrinogen, chitosan and heparin with to enhance the scaffold’s properties [178].

#### 3.2.5. Polyglycolic Acid—PGA

Polyglycolic Acid (PGA) is an FDA-approved linear aliphatic polyester, which is non-toxic and does not induce immunogenicity, it is biodegradable and biocompatible [179]. PGA can be derived from natural resources such as rice, wheat and sweet potato via the process of fermentation and polymerization [180], which makes it an alternative to animal-derived scaffolds. These scaffolds are vastly used because of their tunable degradation rate and easy processing [181]. PGA is a thermoplastic; thus, it can be easily manufactured in accordance with tissue specifications [182]. Its degradation occurs by non-enzymatic hydrolysis, which results in non-toxic metabolites. PGA has a tensile strength of 57 MPa and a Young’s modulus from 6–7 GPa [183]. Nonetheless, PGA and its copolymers, including poly(lactide-co-glycolide) or PLGA—disintegrate relatively fast since their tensile strength decays to half within two weeks [184]. Consequently, like many other polymers, it has been modified to enhance its properties. For example, the use of polyurethane coating has been proven to enhance cell adhesion, growth, and proliferation in PGA scaffolds [185].

These scaffolds were successfully used for the culture of autologous smooth muscle and urothelium, which effectively emulated the function of bladder tissue [186], which makes it a viable option to reproduce similar smooth tissue within OOAC systems.

#### 3.2.6. Polyurethane—PU

Polyurethane scaffolds have gained increasing interest for biomedical applications since they are biocompatible and have advantageous mechanical properties [187]. These synthetic polymers have a distinctive segmented structure, which allows the manipulation of a wide range of properties. The composition and manufacture of the polymer will affect properties such as hydrophilicity and the degradation rate [188].

This biomaterial can be manufacturing using a variety of techniques, which allows a customised production according to the scaffold aim [187].

TPU has been recently used as a scaffold for a Lung-on-a-Chip system due to its biocompatibility, flexibility emulating the mechanical stretching of the in vivo membrane and optical clarity, which easily allows observation and collection of qualitative data. Thus, this material can be used successfully as a biocompatible and versatile scaffold for the growth of complex cellular constructs within OOAC devices, specifically those that required mechanical cues such as cyclic stretching [189]. Novel biodegradable PU elastomers have been developed to act as scaffolds for soft tissues. These incorporate PEG and poly(δ-valerolactone-co-ε-caprolactone) and have shown E = 600 ± 140 kPa [190].

### 3.3. Materials Overview

Table 1 below contains an overview of the materials’ advantages and disadvantages according to the literature described in Section 3 of the paper.

## 4. Manufacturing Techniques

Manufacturing techniques for tissue engineering scaffold production have been developed and optimised for decades. The different characteristics of each of these are vital to produce scaffolds with different mechanical and chemical properties and, alongside material properties, add to their versatility.

Pore size, fibre diameter and architecture are some of the properties that are tunable depending on the technique of manufacture chosen. These play a vital role in the cell viability of different tissues because of the different mechanical requirements for each cell line whilst providing structural integrity and support for the cells and their ECM. Table 2 gives an overview of manufacturing techniques, materials used, pore size range, and a brief overview of the advantages and disadvantages of each method (Section 4.6).

When choosing a suitable manufacturing method pore size and the amount of porosity are important. Pore size will depend on the size of cell type under investigation, porosity is an important factor to consider as it will allow cell media to reach cells embedded in the membrane and cell signalling factors including cytokines, chemokines and growth factors along with nutrient exchange to take place. The pore size chosen will depend on the final use of the device and tissue under investigation; for example, trophoblasts were successfully cultured on two matrices with a pore size of 30 and 39 µm with optimal proliferation seen on the 30 µm membrane. However, fibroblasts have been seen to proliferate on membranes with greater pore size, 100–150 µm depending on porosity [193,194]. The pore size and porosity will further affect the mechanical properties of the materials, too high a porosity and the scaffold will become mechanically unstable. This must be taken into consideration when designing a scaffold for OOAC applications.

### 4.1. Electrospinning

**Figure 2:**
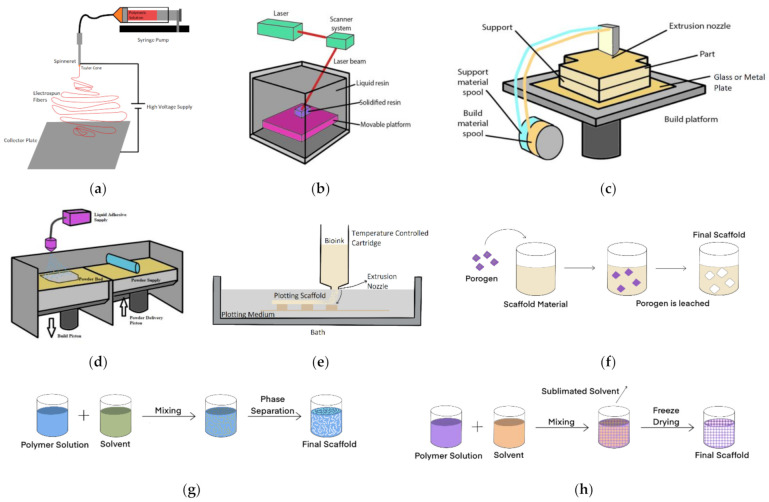
This figure includes schemes of each manufacturing technique manufacturing process. (**a**) Electrospinning Scheme—Section 4.1; (**b**) stereolithography or SLA scheme—Section 4.2.1; (**c**) fused deposition modelling or FDM scheme—Section 4.2.2; (**d**) selective laser sintering or SLS scheme—Section 4.2.3; (**e**) bioplotting scheme—Section 4.2.4; (**f**) salt leaching scheme—Section 4.3; (**g**) phase separation scheme—Section 4.4; (**h**) freeze drying—Section 4.5.

Electrospinning is a common method used to manufacture nanofibre scaffolds [195]. This technique consists of the application of a high voltage to produce an electric field between the needle tip, from which a polymer solution is dispensed and the ground plate where fibres are collected (Figure 2a). The applied voltage induces a charge within the polymeric solution [196]. Once the electrical charges overcome the surface tension of the fluid, a Taylor cone is formed, and a polymeric jet is expelled from the spinneret of the capillary. The solution droplets will be drawn towards the collector plate as it does not go through Rayleigh volatilities [197]. With an increased surface charge density, the fluid jet will turn into fibres as it approaches the collector, producing a mesh of fibres. The diameter of the electrospun product can be controlled by altering the flow rate of the syringe pump, the concentration of the fluid, and the distance between the needle tip and the collector [198]. The increasing diameter of the fibres is associated with the increased average pore size of the scaffold [199]. The high surface area and high porosity inherent to electrospun membranes are beneficial for cell attachment and waste exchange [22]. Electrospinning usually creates a nonwoven fibrous mesh with a pore size from around 3–5 μm. However, it can be manipulated by the fibre orientation and polymer chosen [200].

This technique is straightforward and inexpensive and can be used with a wide variety of polymers to produce viable scaffolds (Table 2). Nevertheless, the polymer must be turned into a solution to allow the electrospinning process, and this requires the use of toxic organic solvents during the manufacturing process, which can be cytotoxic and impact cellular viability [201]. Moreover, the fibres spin randomly, making it hard to control the pore size and structure [202]. Some examples of scaffold materials used in this manufacturing technique are available in Table 2 (Section 4.6).

This technique has been incorporated to create an electrospun PLGA membrane into a Lung-on-a-Chip developed by Yang et al. The membrane was used for co-culture of lung cancer derived epithelial cell, lung fibroblasts and human umbilical vein endothelial cells [191]; the group show a facile sealing method and thus ease of integration into an OOAC system. Chen et al. have developed a novel method to electrospin PCL fibres directly into 3D printed microfluidic channels to culture fibroblasts with a pore size of 113 ± 19 μm showing the diversity of the technique [78].

### 4.2. Three-Dimensional (3D) Printing

There are numerous methods of 3D printing, some involving the deposition of layers, others the solidification of polymers. The shape of the model is predetermined and designed by computer-aided design (CAD) software, together with a slicer software and material choice allows the control of pore size, porosity and architecture [203]. An overview of each 3D Printing technique is available in Table 2 (Section 4.6), along with some material examples and pore size dimensions.

#### 4.2.1. Stereolithography (SLA)

Stereolithography is a 3D printing technique that uses an ultraviolet (UV) LASER to cure an individual layer within a vat of UV curable photopolymer resin (Figure 2b) to create the scaffold [204].

CAD files define the accurate pattern that the UV laser will irradiate. Free radicals are produced upon the excitation of photoinitiator molecules of the UV, which leads to the polymerization of the polymeric scaffold layer. The first layer is used as an adherence platform, which offers support to the scaffold structure. After the process is completed, the model is cured in an oven [205]. This technique allows the production of an array of shapes using different polymers. The resolution of the architecture is dependent on the spot size of the chosen laser and of the elevator layer [22]. Nonetheless, the additional curing necessary to improve the scaffold properties compromise the resolution due to the shrinkage that may occur during postprocessing. Further, there is a limited amount of materials that can polymerise under UV [206]. The benefit of stereolithography over techniques such as electrospinning is that the final scaffold is well defined with known pore size.

Stereolithography has been used in macro tissue engineering, often for bone regeneration, and this can be translated to OOAC technologies looking for similar mechanical properties. Techniques including ceramic stereolithography have become available and used to understand optimal pore size for osteogenesis [207]. SLA has a porosity of 25–100 μm [208]. Being one of the most accurate and with the ability to produce small pore sizes, this could be a useful manufacturing technique for the OOAC field.

#### 4.2.2. Fused Deposition Modelling (FDM)

Fused deposition modelling is a cost-effective and fast technique to produce porous scaffolds [209] with a honeycomb-like structure that creates an interconnected network of channels required for cell and media perfusion [210].

A thermoplastic polymer filament is melted above its glass transition temperature by a small temperature extruder and printed layer by layer (Figure 2c) [22]. This method allows the manipulation of the porosity and structure pattern [211]. Much like the other rapid prototyping methods, FDM requires elevated temperatures, which limit the materials that can be used with this technique [22].

Nonetheless, it is difficult to create scaffolds with a specific porosity and pore size using this technology. Corral, Bagheri, Rojo have been able to optimise the technique to achieve pore sizes around 100–300 μm with PCL [212]. This is a method that has been used successfully in macro tissue engineering and can be translated to OOAC devices with careful control of the deposited filament diameter and pore size. The most readily available material is PCL, which has been mixed with a range of additives for printing scaffolds, including hydroxyapatite, collagen [213], PLA and carbon nanotubes [214]. Fibroblasts have been successfully seeded and grown on scaffolds over a four-week period [215]. FDM has also been successfully used in bone tissue engineering [214].

#### 4.2.3. Selective Laser Sintering (SLS)

Created in 1984 by Carl Deckard [216], selective laser sintering (SLS) has been used for the design and manufacture of the intricate three-dimensional microarchitecture of polymeric, metallic, and ceramic scaffolds, which makes it a largely used fast technique [217].

In this process, a two-dimensional powder layer is printed on top of each other and merged when the binder solution is printed. When the model is removed, any unbounded powder is left behind (Figure 2d). This technology allows the production of scaffolds with high resolution and accuracy and the manipulation of its architecture [218]. Moreover, the complex shape of the scaffold leads to a homogenous cell distribution and emulates the natural ECM. 3D printing scaffolds’ structures range from millimetres to nanometres. The technology tends to be used with materials including hydroxyapatite, calcium carbonate composites and chitosan [219,220]. The technique is again used in bone tissue engineering. One drawback is the high temperature involved in this technology, which limits the number of polymers that can be used to produce a scaffold and requires a high energy input [216]. Degradation of the polymer may also occur—chain scission and oxidation—due to exposure to the laser [221]. Pore size varies according to the design of the scaffolds, nevertheless, studies recorded that PCL membranes can range from 500–800 μm [222].

#### 4.2.4. Bioplotting/Bioprinting

Emerging technology has taken the principle of 3D printing and embedded cells in the process [223]. It takes advantage of computer-aided tissue engineering to build intricate scaffolds and even artificial 3D tissues [224]. A viscous material is ejected into a liquid solution with a similar density (Figure 2e), therefore, the scaffold can be plotted in a 3D structure without the requirement for support structures. This technology allows the use of a variety of different materials such as hydrogels, polymeric solutions, and bioactive polymers with proteins, among many others [225]. The materials have a high water concentration, which allows the creation of cross-linked structures throughout the covalent bonds, which hardens the scaffold [35]. The bioplotter has the ability to dispenses solutions with live cells and the chosen polymer by precisely locating the solution layer-by-layer in a variety of sizes and with high throughput [226].

Historically, the bioplotting downside has been its low resolution, which is a result of the flexibility of the bioinks and the curing of hydrogels. Moreover, this technique makes cells undergo high shear stress when the bioink is extruded [67].

This technology has allowed the fabrication of artificial blood vessels [227] and skin [226]. Nevertheless, this is a costly technology, thus, limiting its use within research [35]. It has been taken up by the OOAC community and been used to develop scaffolds for a Heart-on-a-Chip using endothelial cells plotted with GELMA and cross-linked using UV light; pore size of around 100 μm was shown [228]. Along with cardiac tissue, bioplotting has been used successfully to mimic blood vessels, liver and kidney on a chip [229].

### 4.3. Salt-Leaching

Salt-leaching is a simple technique largely used for scaffold fabrication [230]. The mould is filled with the chosen polymer, which occupies the spaces in between the porogen or salt crystals. To create the scaffold, the polymer will be hardened within the mould, and the porogen or salt will be washed away with a solvent, for example, water or alcohol (Figure 2f). This process allows the creation of pores for cell attachment [231]. Some examples of materials used with this technique are available in Table 2 (Section 4.6).

The pore size of the scaffold can be adjusted according to the amount of porogen/salt and these particle diameters, respectively. Moreover, this method requires a small amount of polymer, which reduces material waste while fabrication, unlike other rapid prototype fabrication methods. Salt leaching scaffolds have a porosity up to 93% and a pore size up to 500 μm. The size can be adjusted by using different porogen with different sizes [232].

The salt-leaching technique cannot control the pore shape and does not reproduce the interconnected channels of pores necessary for media perfusion [233]. Hence, the hybridization of salt-leaching with other manufacturing technologies may provide a solution to these limitations [234].

The technique has been used successfully in macro tissue engineering but is yet to be seen in OOAC applications. As the method tends to be used with hydrogels it is most suitable for engineering soft tissues.

### 4.4. Phase Separation

Phase separation relies on the principle that a homogeneous solution, when under certain conditions, turns thermodynamically unstable, resulting in phase separation thus that the system’s free energy may be lowered [235]. A solvent is used to dissolve the chosen polymer and pored within a mould, which suffers precipitously cooling till the solvent freezes (Figure 2g) [22]. For example, gelatin scaffolds manufactured with this technique have an average pore size of 100 μm [236].

This process creates two phases, one that is polymer-rich and the other which is polymer-poor. The polymer-rich phase will solidify while the latter crystallises [187]. The solvent in the crystallised phase is removed, leaving a porous scaffold structure. The technique does not require the leaching step, however organic solvents used for dissolving the polymer prevent the addition of bioactive molecules, such as RGDs. The small pore size limits the cell types and tissues that can take advantage of a phase separation manufacture [237].

Chen et al. have recently proposed a microporous silk fibroin membrane for OOAC applications, this is yet to be introduced to an OOAC system, pore size is small <2 μm [238].

### 4.5. Freeze Drying

Freeze-drying was firstly used by Whang et al. to manufacture PLGA scaffolding in 1995 [192]. This is a useful technique for the manufacture of porous hydrogels [239]. An organic polymeric solution is poured into a mould and quickly frozen with liquid nitrogen (Figure 2h). Once the polymer is freeze-dried, the pressure is decreased, and the water is sublimated into the air phase, which leads to a porous scaffold structure [240].

The porosity and the pore size can be controlled by adapting the ratio between the water and the chosen polymer in the solution and changing the viscosity of the mix [237]. Moreover, the pore architecture within the scaffold can be regulated by altering the temperature during the process [241]. Pore size decreases when the freezing temperature is diminished. Moreover, studies have shown that the insertion of an annealing step increases pore size by (40%) [242].

The removal of the various rinsing steps necessary in other techniques makes this a good alternative since the solvents can be easily eliminated from the scaffold [192]. Nonetheless, it is a long consuming process requiring elevated levels of energy consumption and the use of cytotoxic solvents used to dissolve the polymer [243].

Freeze drying has been used to produce scaffolds for both soft tissues, cartilage and bone tissue engineering at the macro scale with silk-fibroin, collagen/HA and PCA/PCL/HA, respectively [244,245,246]. It is yet to be integrated into OOAC devices.

### 4.6. Manufacturing Overview

The table below contains an overview of the manufacturing techniques, examples of materials used and pore sizes according to the literature described in Section 4 of the paper. To date, bioplotting/bioprinting has been the most utilised novel scaffold fabrication technique whilst electrospun scaffolds are being more commonly integrated within systems.

## 5. Discussion

Cellular growth and tissue development are intricate processes that are dependent on many factors that can be challenging to control. In cultures attempting to mimic the structure and architecture of tissues, the scaffold must be produced as an initial temporary ECM for cellular support and allow cellular communication and growth while proving a stable structure for the tissue.

Materials and manufacturing techniques are in permanent evolution and development. Even though there are a plethora of components that can be used to produce scaffolds, current state-of-art approaches are far from achieving a scaffold that fits every tissue engineering requirement. Animal-derived materials are largely used since they are like the human tissue ECM and possess growth factors, ECM proteins and hormones, which control cellular growth and tissue formation. Synthetic materials have good mechanical properties, such as elasticity and elastic modulus whilst showing biocompatibility with human cellular tissue.

Current scaffold manufacturing technologies still face challenges that must be tackled. Smaller pore sizes are associated with random architecture in technologies such as electrospinning, salt leaching and phase separation. On the other hand, techniques that have a high resolution of scaffold architecture are associated with pore sizes on the mm scale. Salt leaching, electrospinning and phase separation techniques involve the use of toxic solvents to dissolve the polymer, and this can be harmful to cells. Salt leaching does not create a wide interconnected pore network, which would limit the flow of medium with fresh nutrients and the expulsion of cellular waste. To solve these challenges, researchers have created hybrid polymeric scaffolds by using a combination of different techniques and by combining additives that increase cellular viability.

Moreover, there is a lack of standards when it comes to the use of scaffolds for specific cell types, with studies using different materials, which makes it hard to compare the resultant data from different research groups. Thus, it is critical to test different scaffolds using specific cell types that have the same requirements and analyse the data using the same protocols to produce a single optimal scaffold that optimally emulates in vivo microenvironment. The creation of standards for the most used cell types within the OOAC research is a novelty that will progress the research field.

Various studies have combined, for example, PCL with different naturally-derived compounds such as gelatin, collagen, chitosan and RGD, among others, thus that the properties of this scaffold can be optimised for its specific purpose. PLA is another example of a polymer that has been combined with hydroxyapatite, GE, chitosan, among others. This has become the current practice since cells thrive on these hybrid scaffolds. Current materials used for scaffolding in the OOAC research field consist of PDMS and PCL, which have little or slow degradation rates and, therefore, compete for space with the cellular tissue.

Traditional techniques used to manufacture scaffolds do not allow parameters such as pore size and porosity to be altered once manufactured. The creation of tunable scaffolds is the next step in the innovation of tissue engineering membranes. Biological tissues are extremely dynamic, therefore, “smart” scaffolds that can adapt to the cells’ requirements are needed to create a suitable microenvironment for cell growth for specific tissues.

## 6. Conclusions

In this paper, we reviewed the different materials and manufacturing techniques that could be used to produce scaffolds for the OOAC research field.

Scaffold manufacture research and tissue engineering have been greatly improved in the past decade with the creation of new technologies. Nevertheless, the current materials and manufacturing techniques still require significant research.

From the literature, it is evident:

- To move from animal-derived products, further research and development using synthetically derived products are required to achieve the same quality and reproducibility of data. The use of RGD or similar anchoring proteins increases cell viability in the tissue, enhancing the properties of synthetic materials.
- Manufacturing techniques can be used to manipulate scaffold properties according to the requirements of the specific tissue making them dynamically tunable. Combining different techniques and using the right materials is the key to OOAC scaffold standardisation.
- Current OOAC systems use their version of scaffolds, which makes data hard to compare across research groups. This presents a major obstacle to the use of these devices as pre-clinical models since they are not regulated. Hence, the creation of Standards approved by entities such as the European Centre for the Validation of Alternative Methods (ECVAM) is crucial for the approval of these systems as validated models. Researchers from both academia and industry in collaboration with regulators could use standards such as ISO/TS 21560:2020 (general requirements of tissue engineering medical products) as a starting point to close the gap in regulation within the OOAC research field.

There is still a lot of work to be conducted to create optimal scaffolds. Overcoming the current scaffold limitations will lead to more reliable and reproducible OOAC data.

As a final point, this review paper aims to provide some guidance for researchers in the OOAC research field by encouraging further research in the development of scaffold standards for specific cellular tissues that allow emulation of the in vivo environment and reducing the cost involved.

## Figures and Tables

**Table 1:** Materials overview.

Material	Advantages	Disadvantages	Examples of OOAC	References
Matrigel	Promotes cell adherence. Extremely biocompatible. Similar mechanical properties as natural ECM.	Batch to batch composition variability may affect results.	Liver-on-a-Chip	[10,15,16,80,81,82,83,85,86,89]
Collagen	Most predominant protein in mammals’ ECM. Controls morphology, adhesion, and differentiation. Good permeability.	Lacks mechanical strength and structural stability when hydrated.	Gut-on-a-Chip	[90,91,92,93,94,95,96,98,99,101,102]
Chitosan	Biocompatible. Biodegradable. Permeation enhanced.	Mechanically week and unstable.	Vascular microfluidic device	[103,104,105,106,107,108,109]
Alginate	Reduced production cost. Mechanical Strength can be adjusted depending on molecular weight.	Purity will affect biocompatibility. It will not naturally degrade in mammal derived tissues. Poor cell adhesion.	Microfluidic devices using cardiac tissue, liver and hepatocytes.	[111,112,113,114,115,116,117,118,120,121,122,123]
Cellulose	Insoluble in water. High mechanical strength. Allows water retention. Biocompatible. Optical transparent.	Degraded by microbial and fungal enzymatic activity.	Microfluidic device using lung cancer cells.	[124,125,126,127,129,130,131,132]
Gelatin	Biocompatible. Biodegradable. Good water-solubility. Low cost.	Animal-derived.	Microfluidic devices emulating vascular environments.	[134,135,136,137,139,140,141]
Poly-(Ɛ-caprolactone)—PCL	Low cost. Compatible with many manufacturing techniques. Good mechanical properties.	Slow biodegradability rate.	Microfluidic devices to emulate the myocardium and vascular environments.	[142,143,144,145,146,147,148,149,150,151,152,153]
Poly-(dimethyl-siloxane)—PDMS	Biocompatible. Optical transparent. High flexibility adequate for cyclic stretching.	Non-degradable. Hydrophobic. Absorbs small molecules.	Lung-on-a-Chip Kidney-on-a-Chip Brain-on-a-Chip	[7,13,154,155,156,157,158,159,160,161,162,163,164,165]
Polylactic Acid—PLA	Biodegradable. Mechanical properties are tunable.	Not suitable for OOAC systems with high mechanical strain.	Cell culture chips with MSCs	[49,166,167,168,169,170,171]
Polyglycolic Acid—PGA	Biodegradable. Biocompatible.	High degradation rate.	3D cultures of autologous smooth muscle and urothelium	[179,180,181,182,183,184,185,186]
Poly(lactide-co-glycolide)—PLGA	Biodegradable. Biocompatible. High Porosity. High Mechanical Strength.	High degradation rate.	Lung-on-a-Chip Blood-Brain Barrier Vascular Network Scaffold	[37,42,152,184,191,192]
Polyurethane—PU	Hydrophilic. Biocompatible. Biodegradable. Compatible with many manufacturing techniques.		Lung-on-a-Chip	[187,188,189]

**Table 2:** Manufacturing Techniques overview.

Manufacturing Technique	Advantages	Disadvantages	Examples of Scaffolds Produced	Pore Size	References
Electrospinning	High surface area. High porosity. Simple and inexpensive solutions can be used. Wide range of polymers.	Toxic organic solvents are used.	PCL	~3–5 μm.	[22,195,196,197,199,200,201,202]
			Gelatin		
			PCL/Collagen Chitosan		
SLA	Controlled resolution.	Final resolution may be compromised by shrinkage in colling process.	PCL	25–100 μm.	[22,204,205,206]
			Calcium phosphate (CaeP)/poly (hydroxybutyrate-co-hydroxyvalerate) (PHBV)		
FDM	Precise deposition of thin layers of polymers.	Elevated temperature that limits material choice.	PCL	100–300 μm.	[22,209,210,211,212]
SLS	Fast and cost-effective. Does not require the use of organic solvents.	Elevated temperature requires high energy input. Degradation of the material may occur.	PCL	Minimum around 400 μm.	[216,217,221,222,247]
			Poly(ethylene glycol)/poly(D,L-Lactide) hydrogel		
Bioplotting	High resolution for an extrusion system. Various materials can be used during the print.	Limited geometry designing. High-cost technology.	Poly(lactides) PCL Poly(lactide-co-glycolide) Chitosan		[35,223,224,225,226]
			Gelatin		
Salt-leaching	Small amount of polymer needed. Does not require large machinery. Low-cost.	Interpore opening and pore size are not controllable.	Sodium Chloride as porogen.	Up to 500 μm, dependent on porogen size.	[230,231,232,233,234]
Phase Separation	Harsh chemical solvents are not needed. Lower fabrication time.	Use of organic solvents (e.g., ethanol or methanol) inhibits incorporation of bioactive molecules. Small pores.	Gelatin/silica hydrogels.	~100 μm Depends on porogen size.	[22,187,235,236,237]
Freeze Drying	No rinsing steps.	Reduced heterogeneous freezing may occur.	PLGA	~85–325 μm.	[192,237,239,240,241,242,243]

## Data Availability

Not applicable.
